# Discovery and Heterologous Expression of the Soil Metagenome-Derived Lasso Peptide Metanodin with an Unprecedented Ring Structure

**DOI:** 10.1021/acs.jnatprod.5c00970

**Published:** 2025-10-27

**Authors:** Timo Negri, Giovanni Andrea Vitale, Martina Adamek, Caner Bağcı, Julian D. Hegemann, Daniel Petras, Chambers C. Hughes, Nadine Ziemert

**Affiliations:** † Translational Genome Mining for Natural Products, Interfaculty Institute of Microbiology and Infection Medicine (IMIT), 234487 University of Tübingen, Auf der Morgenstelle 24, 72076 Tübingen, Germany; ‡ German Centre for Infection Research (DZIF), Partner Site Tübingen, 72076 Tübingen, Germany; ⊥ Functional Metabolomics Laboratory, Interfaculty Institute of Microbiology and Infection Medicine (IMIT), University of Tübingen, Auf der Morgenstelle 24, 72076 Tübingen, Germany; ¶ Department of Microbial Bioactive Compounds, Interfaculty Institute of Microbiology and Infection Medicine (IMIT), University of Tübingen, Auf der Morgenstelle 28, 72076 Tübingen, Germany; ∥ Institute for Bioinformatics and Medical Informatics (IBMI), University of Tübingen, Sand 14, 72076 Tübingen, Germany; ◆ Institute of Pharmaceutical Biology, Technische Universität Braunschweig, 38106 Braunschweig, Germany; ○ Center of Pharmaceutical Engineering (PVZ), Technische Universität Braunschweig, 38106 Braunschweig, Germany; ⬠ Department of Biochemistry, University of California Riverside, Riverside, California 92507, United States; ◪ Cluster of Excellence EXC 2124: Controlling Microbes to Fight Infection, University of Tübingen, 72076 Tübingen, Germany

## Abstract

Culture-independent metagenomic approaches have proven to be effective tools for identifying previously hidden biosynthetic gene clusters (BGCs) encoding novel natural products with potential medical relevance. However, producing these compounds remains challenging as metagenomic BGCs often originate from organisms phylogenetically distant from available heterologous hosts. Lasso peptides, a subclass of ribosomally synthesized and post-translationally modified peptide (RiPP) natural products, exhibit diverse bioactivities, yet no lasso peptide has previously been discovered directly from a metagenome. Here, we report the discovery and heterologous expression of the first soil metagenome-derived lasso peptide. Expression of its biosynthetic gene cluster in *Escherichia coli*, followed by mass spectrometry analysis, strongly supported the predicted amino acid sequence and lasso structure of the peptide. Notably, this lasso peptide is the first to feature asparagine as the ring-forming residue at position one. Taxonomic analysis of the corresponding BGC identified an uncultivated member of the *Steroidobacterales* family (*Gammaproteobacteria*) as the closest known relative of the potential native host. These findings underscore the potential of metagenomic genome mining to reveal structurally novel RiPPs and to expand our understanding of the natural diversity of lasso peptides.

## Graphical abstract

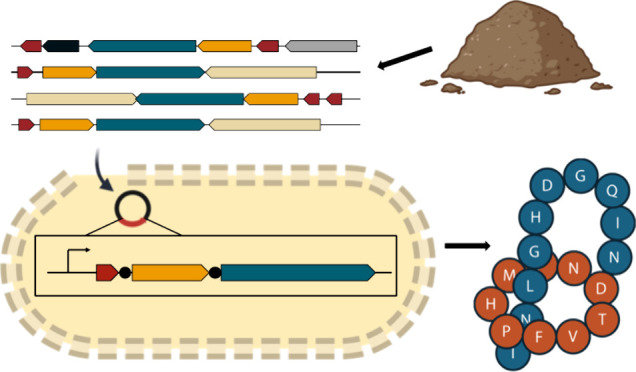

Soils harbor one of the most diverse microbial ecosystems on Earth, containing thousands of microbial species per gram, the majority of which remain uncultivated. Genomic studies have revealed that these microorganisms encode an enormous diversity of biosynthetic gene clusters (BGCs), many of which are predicted to produce structurally novel and potentially bioactive natural products. The disparity in compound structural diversity between environmental metagenomes and culture collections underscores the vast biosynthetic “dark matter” hidden in uncultured microbes.^1,2^

Traditional natural product discovery has relied on cultivation-based methods, which have successfully yielded a broad spectrum of antibiotics and other bioactive compounds, particularly from soil-dwelling *Actinomycetota*. However, the rate of new compound discovery has slowed in recent decades,^3^ despite clear evidence that even well-studied strains harbor far more BGCs than the number of known metabolites they produce.^4^ While advances in microbial cultivation^5–7^ have extended access to previously elusive taxa, the majority of environmental microbial diversity remains inaccessible with standard laboratory techniques.^8–11^

Culture-independent, sequence-based metagenomic approaches offer a powerful alternative to natural product discovery. By extracting and sequencing environmental DNA, these methods bypass the need for cultivation and allow for the identification of BGCs directly from microbial communities.^2,12^ However, accessing the actual compounds remains challenging. Many metagenomic BGCs derive from phylogenetically distant organisms, complicating their functional expression in standard heterologous hosts such as *Escherichia coli* (*E. coli*).^13,14^ Nonetheless, several successful examples of metagenome-derived natural products demonstrate the promise of this approach.^13,15^

Lasso peptides are a structurally unique and pharmacologically attractive subclass of ribosomally synthesized and post-translationally modified peptides (RiPPs). Their characteristic threaded conformation, a C-terminal tail threaded through an N-terminal macrolactam ring, endows them with high stability and a range of bioactivities, including antibacterial, antiviral, and anticancer effects.^16–18^ Biosynthesis of lasso peptides typically involves three core genes: a precursor peptide gene (A), a leader peptidase (B, or split into B1 and B2), and an ATP-dependent macrolactam synthetase (C). Additional genes, such as D, encoding an ABC transporter, are sometimes present and may support self-resistance or secretion.^19,20^ Despite increasing interest, no lasso peptide has been discovered directly from a metagenome.

Here, we report the discovery and heterologous production of the first lasso peptide derived from a soil metagenome. While we initially recovered multiple lasso peptide BGCs, only one could be successfully expressed in *E. coli*, yielding a novel lasso peptide whose structure was strongly supported by mass spectrometry. Remarkably, this peptide exhibits asparagine as the first residue of the macrolactam ring, a feature predicted but never experimentally observed before. Taxonomic analysis indicated that the gene cluster originated from an uncultivated member of the *Steroidobacterales* with no close relatives among sequenced cultured species. Our findings illustrate how metagenomic genome mining and heterologous expression enable access to novel metabolites encoded in uncultivated soil microbes and, in turn, can broaden the scope of even well studied natural product classes.

## Results and Discussion

### Identification of Novel Metagenomic Lasso Peptide BGCs

We previously developed a strategy for the rapid identification and recovery of biosynthetic gene clusters from soil metagenomes.^14^ In this follow-up study, we aimed to determine the heterologous expression of such metagenomic BGCs. We focused on lasso peptides because these special peptides exhibit a variety of therapeutically relevant bioactivities and are characterized by high stability due to their unique structure. As a source for novel lasso peptide BGCs, we used the previously generated sequencing data of our metagenomic fosmid library as well as sequencing data of its corresponding high molecular weight (HMW) metagenomic DNA isolated from a soil sample of the Schönbuch forest (Germany).^14^ Lasso peptide BGCs are typically smaller than other natural product BGCs such as those encoding nonribosomal peptide synthetases (NRPSs) or polyketide synthases (PKs). Therefore, we filtered the contigs of our metagenomic fosmid library for smaller sizes, e.g., contigs greater than 5 kb, as opposed to the previously reported 40 kb, which allowed for the detection of a greater number of lasso peptide BGCs. The antiSMASH^21^ analysis identified 94 lasso peptide BGCs on contigs greater than 5 kb, none of which showed similarity to a known gene cluster within the MIBiG database^22^ using antiSMASH’s “KnownClusterBlast” function. These findings are consistent with other metagenomic studies in this field, which have repeatedly reported biosynthetic novelty in soils.^1,10,12,23^

### Prioritization and Classification of Metagenomic Lasso Peptide BGCs for Heterologous Expression

To streamline the production of metagenomic lasso peptides, we aimed to simultaneously recover and express several candidate BGCs in *E. coli*. We prioritized clusters based on the following criteria. First, we selected BGCs in which the core biosynthetic genes were colinear (i.e., in the same orientation), enabling smooth amplification and cloning. Using the strategy described in our previous study, we identified the minimal biosynthetic genes (A, B, and C) as well as additional lasso peptide-associated genes (Supplemental Tables S1 and S2). In cases where antiSMASH failed to annotate an A gene, such as in BGC 89, we performed manual identification based on size and conserved features. To evaluate structural diversity across different BGC architectures, we grouped the selected clusters into two categories based on gene organization.

**Group I** contained BGCs with a classical proteobacterial ABC architecture. Notably, three of these lacked the canonical D gene encoding an ABC transporter and instead included an inversely oriented gene predicted to encode a peptidase, a variation previously reported in some lasso peptide clusters.^24–26^ **Group II** comprised BGCs with nonproteobacterial architecture, featuring split B genes (B1/B2), additional putative tailoring enzymes, and in three cases, intermediating prolyl oligopeptidase genes between the core biosynthetic genes. We decided to recover a subset of BGCs from each group to assess which of the two is the most promising in terms of production success. Hence, we selected BGCs 38, 52, 54, and 89 from Group I (Figure 1A) and BGCs 364, 468, 8976, and 9882 from Group II (Figure 1B).

**Figure 1:**
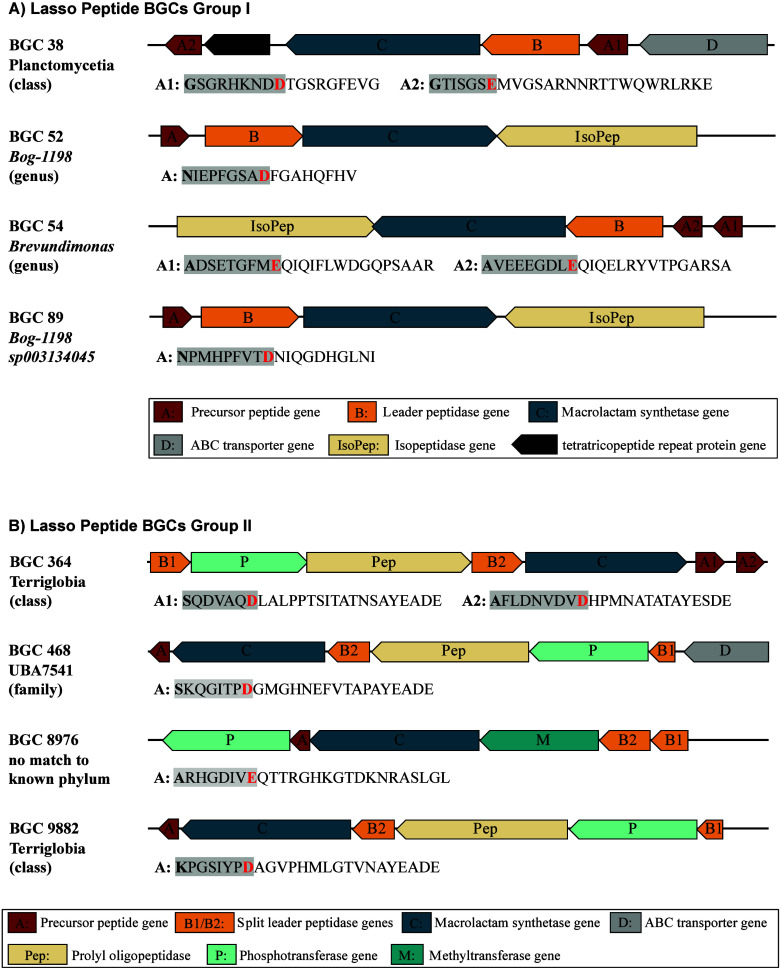
Annotated metagenomic lasso peptide BGCs were prioritized for heterologous expression. Predicted producer organism for each BGC determined to the lowest common ancestor. For each BGC putative core peptide sequences are shown. Amino acids forming the isopeptide bond are highlighted in bold and red. Amino acids forming the ring are highlighted with a gray box. (A) Lasso peptide BGCs with classical proteobacterial ABC cluster organization. (B) Lasso peptide BGCs with nonproteobacterial architectures, featuring split B genes (B1/B2) and additional putative tailoring enzymes.

### Refactoring of Lasso Peptide BGCs for Heterologous Expression in *E. coli*

To improve the likelihood of successful compound production, we refactored each prioritized lasso peptide BGC before cloning. For Group I BGCs (classical architecture), we amplified genes A, B, and C and included the D gene (encoding a putative ABC transporter) in the case of BGC 38. The gene clusters were then introduced into a plasmid downstream of an inducible T7 promoter. Genes predicted to encode peptidases were deliberately excluded to avoid degradation of the lasso peptide, as lasso peptide isopeptidases can cleave the macrolactam ring and linearize the compound.^27–29^ To further enhance expression, we replaced the intergenic regions between the A and B genes with an *E. coli* optimized ribosome binding site (RBS) in all constructs where no clear native RBS was apparent. This strategy has already proved effective in previous studies, where *E. coli* was used as a heterologous host for lasso peptide production.^24,25,30^ For BGC 89, the intergenic region between the B and C genes was also replaced with the RBS. The final construct for BGC 89, including RBS design and promoter arrangement, is shown in Figure 2. For Group II BGCs (nonproteobacterial architecture), we amplified genes A, B1, B2, and C, along with annotated tailoring/modification genes, and excluded the D gene in the case of BGC 468. As with Group I, peptidase genes were excluded, and the optimized RBS was inserted upstream of the B1 gene. These constructs were confirmed by sequencing and subsequently transferred to *E. coli* for peptide production and characterization.

**Figure 2:**
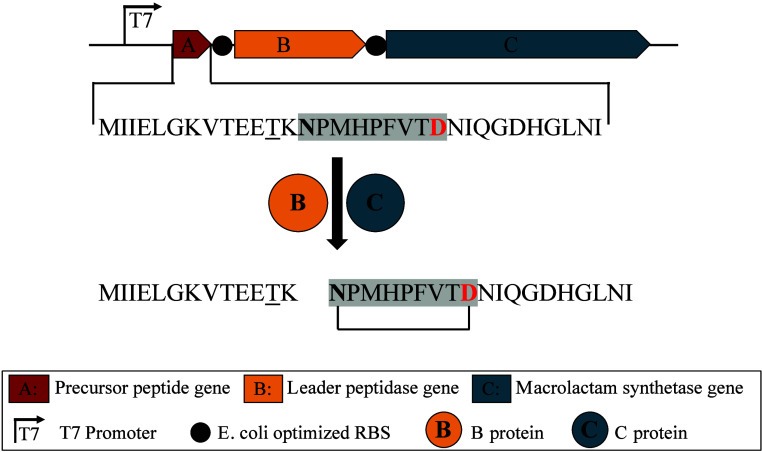
Refactoring and cloning of the lasso peptide BGC 89 under the control of the T7 promoter. Intergenic regions between genes A and B, as well as between B and C, were replaced with an *E. coli* optimized RBS. The translated amino acid sequence of gene A of the lasso peptide BGC 89 is shown. The conserved threonine at position −2 (relative to the first ring-forming amino acid) is underlined. Amino acids forming the isopeptide bond are highlighted in bold and red. Amino acids forming the ring are highlighted with a gray box.

### Discovery of the Novel Lasso Peptide Metanodin from a Soil Metagenome via Heterologous Expression and Mass Spectrometry

To evaluate production for the refactored lasso peptide BGCs, all constructs were transferred to *E. coli*, and cluster expression was conducted in M9 minimal medium. Cell pellets and supernatants were extracted separately and analyzed by high-performance liquid chromatography–mass spectrometry (HPLC–MS). We screened the resulting data for ions corresponding to the masses of the predicted lasso peptides by extracting the respective extracted ion chromatograms (EIC). Only for the strain carrying BGC 89 did we detect a peak consistent with the expected lasso peptide product, while no product peaks were identified in the other cases.

Interestingly, the corresponding A gene in BGC 89 was not annotated by antiSMASH but, instead, labeled as an “other gene”. Manual inspection revealed that its size was comparable to that of known precursor peptide genes, and the translated amino acid sequence (Figure 2) exhibited several features typical of lasso peptides, alongside deviations from known motifs. While the sequence contained a suitable aspartate residue to form the macrolactam ring, none of the experimentally observed N-terminal residues (such as glycine, cysteine, serine, or alanine)^17,31^ were present at an appropriate distance. Instead, we hypothesized that the asparagine residue might serve as the first ring-forming residue. This assignment was consistent with the expected ring size and supported by the presence of a threonine two residues upstream, a motif highly conserved in lasso peptide precursors.^30^ While asparagine has not previously been reported at this position, its small, polar, uncharged side chain may still permit efficient ring formation. Compared to smaller residues, such as glycine or alanine, the amide group of asparagine could potentially influence macrolactam formation, enzyme–substrate interactions, or the structural dynamics of the final peptide. Whether this feature affects the ring strain, protease resistance, or overall stability remains to be determined.

In the MS data, a peak corresponding to the predicted lasso peptide (*m*/*z* [M+2H]^2+^ 1050.9996) was detected exclusively in the pellet extract from the BGC 89-expressing strain and not in the negative *E. coli* control (Figure 3A, corresponding total ion chromatogram in Supplementary Figure S1). In addition to the [M+2H]^2+^ ion, the [M+3H]^3+^ ion with *m*/*z* 701.0022 was also observed (Figure 3B). We named this lasso peptide metanodin in reference to its metagenomic origin.

**Figure 3:**
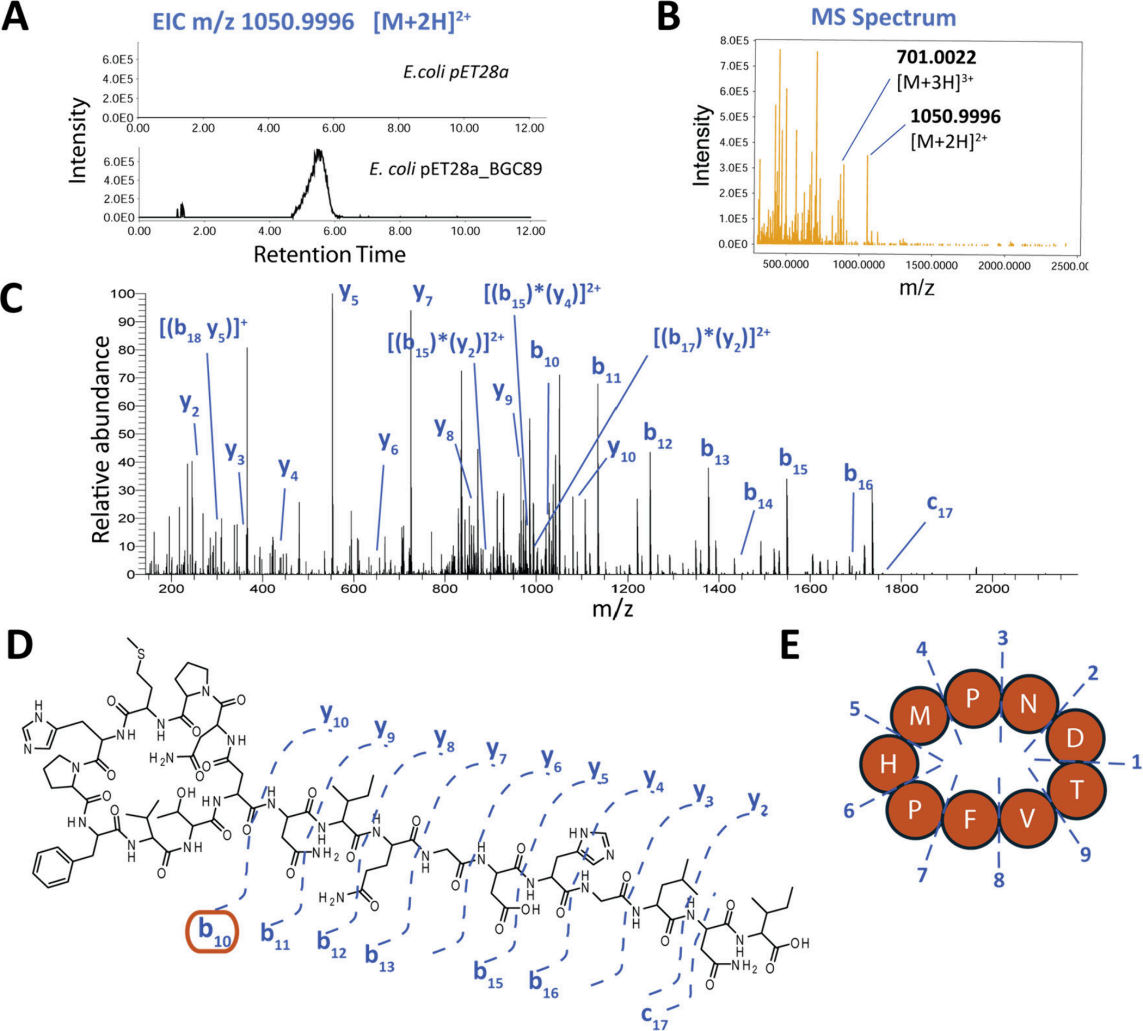
Mass spectrometry and HCD analyses confirm the predicted sequence and structure of the BGC 89-encoded lasso peptide. (A) Extracted ion chromatogram (EIC) of *m*/*z* 1050.9996 (±5 ppm) comparing control *E. coli* (upper graph) and the BGC 89-expressing strain (lower graph). (B) A distinct peak is observed in the BGC 89-expressing strain. The corresponding MS spectrum shows the [M+2H]^2+^ ion at *m*/*z* 1050.9996 and the [M+3H]^3+^ ion at *m*/*z* 701.0022 in accordance with the predicted lasso peptide molecular formula. (C) Targeted collision-induced dissociation of [M+2H]^2+^ was performed at multiple energies, generating MS/MS spectra, which were annotated for structural validation of the sequence and the interlocked fragments presented in Figure 4. (D) The predicted structure was strongly supported by the nearly complete annotation of b- and y-type fragment ions corresponding to the C-terminal region. (E) The macrolactam ring (fragment b_10_) exhibits characteristic cyclic peptide fragmentation with random ring opening events yielding nine distinct linear forms, each producing respective diagnostic b fragments (Supplementary Figure S2).

To confirm the predicted primary structure, the [M+2H]^2+^ ion was subjected to Higher energy Collisional Dissociation (HCD) at multiple collision energies. The resulting MS/MS spectra displayed a nearly complete b- and y-type ion series corresponding to the linear tail of the peptide (NIQG­DHGLNI) with observed b_10_–b_13_, b_15_, b_16_, and y_2_–y_10_ ions (Figure 3C,D), thus confirming the predicted sequence. While cyclic peptides are generally refractory to fragmentation, HCD is particularly effective at cleaving constrained peptide rings. In such cases, random ring openings generate multiple linearized variants, each producing characteristic fragment ions. Fragmentation of the metanodin macrolactam ring (initiated at b_10_) yielded the expected nine distinct linearized peptides (1–9) and a total of 35 diagnostic b-ions, fully supporting the predicted macrolactam ring sequence (Figure 3E and Supplementary Figure S2).

These findings highlight two important considerations for future lasso peptide discovery. First, precursor peptide genes may still be overlooked by automated genome mining tools and may require manual annotation, especially in the context of metagenomic data sets as these might contain novel lasso peptide subtypes. Second, strict reliance on established sequence motifs may result in missed discoveries. As shown here, BGCs from phylogenetically distant, uncultured organisms can encode lasso peptides with previously unreported structural features, including noncanonical ring-forming residues.

Their unique threaded conformation makes lasso peptides more stable and resistant to proteolytic degradation. This comes from their mechanically interlocked structure, and this feature is also reflected in their unique MS/MS fragmentation behavior, where characteristic interlocked b/y fragment pairs can be observed, distinguishing them from unthreaded or branched-cyclic analogues.^32–34^ These [(b_i_)*­(y_j_)] species are especially visible in lasso peptides with long loop regions (≥5 residues) and arise when two bond cleavages occur within that region. As a result, the b- and y- type ions produced by fragmentation remain noncovalently associated, as the C-terminal tail remains sterically trapped inside the macrolactam ring, generating the characteristic “interlocked” ion pairs that serve as strong evidence for a threaded conformation. In this specific case, the metanodin fragmentation spectrum featured three interlocked fragments [(b_17_)*­(y_2_)]^2+^, [(b_15_)*­(y_4_)]^2+^, and [(b_15_)*­(y_2_)]^2+^, the latter detected alongside the complementary internal fragment [(b_18_y_5_)]^+^ (Figure 4 and Figure 3C). These findings strongly support the predicted threaded lasso conformation of metanodin.

**Figure 4:**
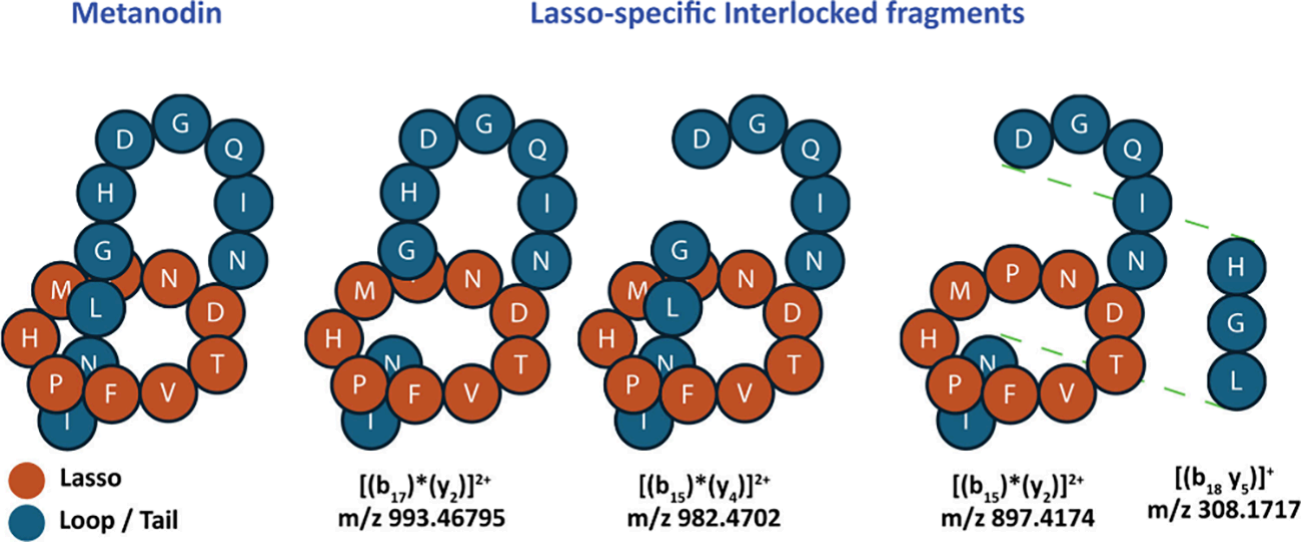
The presence of distinct interlocked fragments is consistent with the threaded lasso conformation of metanodin. The observed metanodin-specific [(b_i_)*­(y_j_)] interlocked species were [(b_17_)*­(y_2_)]^2+^, [(b_15_)*­(y_4_)]^2+^, and [(b_15_)*­(y_2_)]^2+^, the latter being detected alongside the complementary fragment [(b_18_ y_5_)]^+^, thus confirming the predicted lasso conformation.

The amounts of metanodin heterologously produced in *E. coli* yielded sufficient material for a full MS-based structure confirmation, yet production levels remained too low for both peptide isolation and bioactivity testing. Further optimization, including promoter engineering or testing of alternative expression hosts, may help to improve yields and enable downstream characterization.

### Environmental Distribution and Phylogenetic Origin of the BGC

To investigate whether the metanodin BGC or other closely related clusters occur elsewhere in the environment, we analyzed the corresponding contig using BGC Atlas,^35^ a tool for exploring global BGC diversity across metagenomic data sets. The analysis identified a match to gene cluster family (GCF) 1167 with a cosine-like distance of approximately 0.3. Interestingly, although BGC 89 originated from a soil sample, most members of GCF 1167 were found in aquatic marine environments and are broadly distributed across diverse biomes.

We then assessed the phylogenetic origin of the BGC using the Genome Taxonomy Database (GTDB).^36^ The closest match was a metagenome-assembled genome (MAG) from permafrost soil in Sweden, classified as *Bog-1198 sp003134045*, within the *Steroidobacteraceae* family (order *Steroidobacterales*, class *Gammaproteobacteria*). Given that *E. coli* also belongs to *Gammaproteobacteria*, it may offer a relatively compatible host background, which is in line with previous findings.^37^ However, the low production levels observed suggest that further optimization, or the use of alternative expression systems, may be necessary.

Alternative hosts such as *Burkholderia* spp. have been reported to support efficient lasso peptide production,^38–40^ particularly for BGCs originating from *Betaproteobacteria*, the same class to which *Burkholderia* belongs. While our BGC derives from a different lineage within the *Proteobacteria* phylum, testing the expression of the other recovered metagenomic BGCs in *Burkholderia* or other well-established RiPP production hosts may help uncover peptides not detected in *E. coli*.

To evaluate the relation of BGC 89 to available clusters within the NCBI database, we performed a cblaster^41^ analysis to identify homologous BGCs. For visualization with clinker,^42^ we selected the five top-ranking cblaster hits and included the closest match *Bog-1198 sp003134045* (Figure 5A). The latter showed the highest level of similarity (B-gene: 53.50%; C-gene: 56.54%; isopeptidase-gene: 56.70%), followed by a hit with a lasso peptide BGC from a MAG of a Steroidobacteraceae bacterium (B-gene: 41.83%; C-gene: 45.57%; isopeptidase-gene: 44.12%). Notably, these results show that even the two best matches exhibited comparatively low similarities. Additionally, the BGCs identified among the other hits also displayed low similarity to each other, suggesting a high degree of structural diversity among the corresponding lasso peptides. To assess the similarity between metanodin and the predicted lasso peptides from the two closest matches, the corresponding contigs were analyzed using antiSMASH to identify the precursor peptides, which was successful only for the second-best match. The corresponding amino acid sequence was subsequently compared to that of metanodin. While a conserved threonine at the penultimate position and a shared C-terminal residue in the leader peptides were found, no significant similarity between the core regions of the peptides was observed (Figure 5B). Notably, this also applied to the ring-forming asparagine residue in metanodin as the corresponding position in the second closest match was occupied by a glutamine.

**Figure 5:**
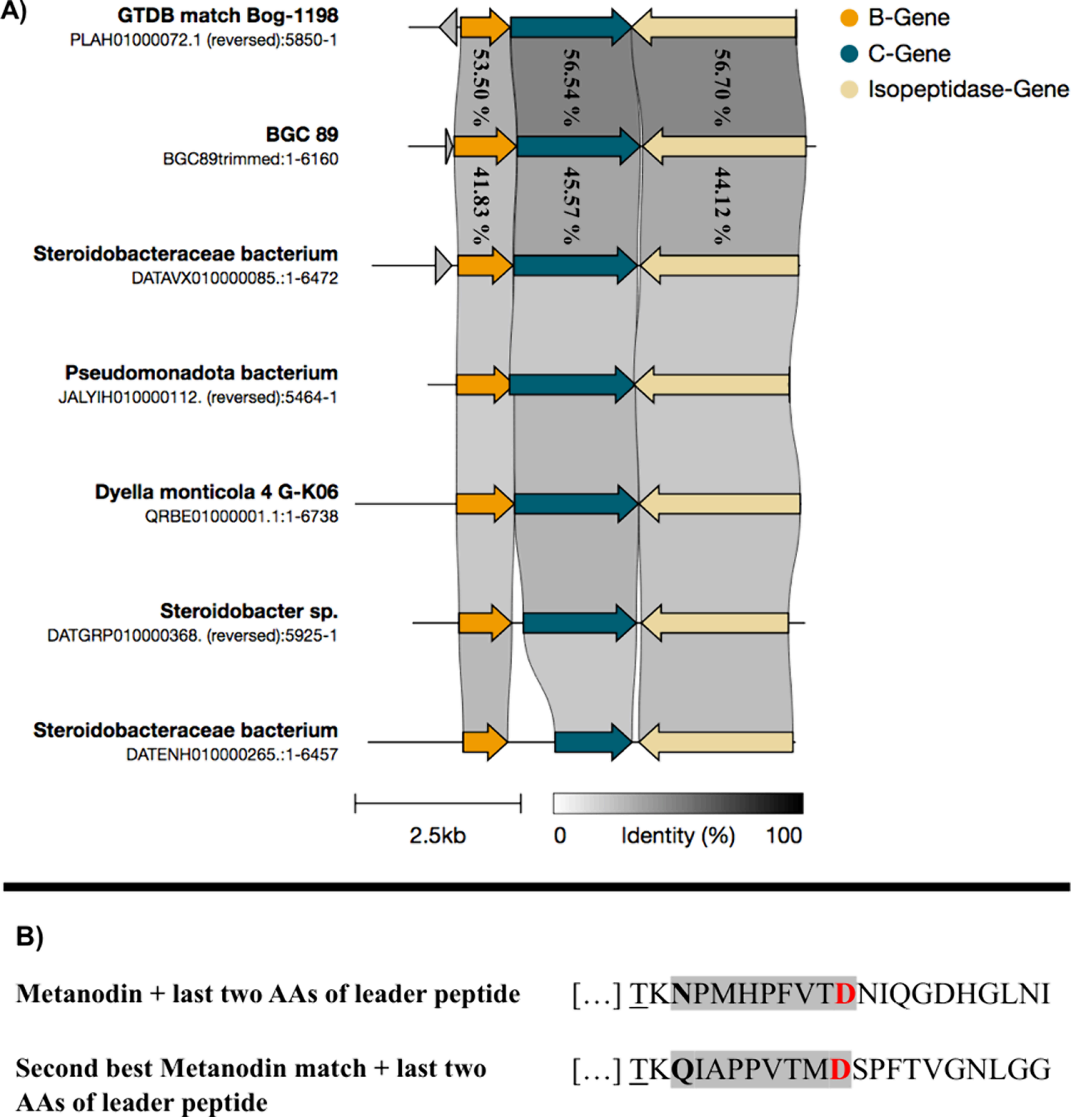
Environmental and taxonomic origin of the metanodin BGC. (A) Relation of BGC 89 to available clusters within the NCBI database and the closest match within the GTDB database. Shown are similarities between BGCs of the five top-ranking cblaster hits along with the closest match within the GTDB database, *Bog-1198 sp003134045*, and BGC 89. The exact percent identity values are only shown for the two best BGC 89 matches. (B) Amino acid sequence of metanodin along with the predicted lasso peptide sequence from the second closest match. The conserved threonine residue at position −2 relative to the first ring-forming amino acid is underlined. Amino acids forming the isopeptide bond are highlighted in bold and red. Amino acids forming the ring are highlighted with a gray box.

These findings further highlight the value of metagenomic approaches for uncovering BGCs from yet-uncultured microbial lineages that may encode structurally divergent natural products. Accessing such BGCs not only expands our view of natural product diversity and distribution but also opens new avenues for discovering molecules with previously unseen structural features and biological targets.

## Conclusions

In this study, we report the discovery and heterologous production of a structurally novel lasso peptide from a soil metagenome, the first of its kind identified through a culture-independent approach. Manual curation of the precursor gene, combined with targeted refactoring and MS/MS analysis, enabled the confirmation of a lasso peptide featuring asparagine as the first ring-forming residue, a previously unobserved motif. The gene cluster originates from a phylogenetically distant, uncultivated member of the *Steroidobacterales* family, underscoring the power of metagenomic strategies to access previously untapped biosynthetic diversity.

Although metagenomic studies and discovery pipelines have been successfully applied to other classes of natural products,^43–45^ including polyketides and nonribosomal peptides, this study demonstrates their feasibility for lasso peptides, which present distinct biosynthetic and expression challenges. In our case, only one out of eight prioritized clusters yielded a detectable product, and production levels remained too low for further characterization, highlighting current limitations in accessing the functional potential of metagenomic BGCs. Addressing these challenges will require improved refactoring strategies, alternative host systems, and emerging technologies such as cell-free expression^46^ or synthetic regulation. Nonetheless, our findings provide a proof-of-concept that lasso peptides from uncultivated microbes can be discovered and expressed using genome-guided approaches, paving the way for future efforts to unlock the full structural and functional diversity of RiPPs from environmental microbiomes.

## Experimental Section

### General Experimental Procedures

LC-MS/MS analysis was performed by injecting 5 μL of sample into an LC-MS/MS system composed of a Vanquish UHPLC and a Q Exactive HF mass spectrometer equipped with a heated electrospray ionization (HESI) source. The latter was operated with the auxiliary gas temperature = 400 °C, flow = 12 arbitrary units (AU), sheath gas flow rate = 50 AU, and sweep gas flow = 1 AU. The targeted fragmentation experiment was carried out in positive ion mode with the following parameters: capillary voltage = 3.5 kV; drying temperature = 250 °C. The analysis was performed on a mass range set to 200–2500 *m*/*z* with a resolution of 120,000. Targeted fragmentation was performed using an isolation window of 2 Da and AGC target = 2 × 10^5^ with a resolution of 45,000 at the following normalized collision energies (NCEs): 15, 25, 35, and 45. For the chromatographic separation, an EVO C-18 column (2.6 μm, 100 Å, 50 mm × 2.1 mm) was employed. As mobile phases, phases A (H_2_O + 0.1% formic acid) and B (ACN + 0.1% formic acid) were used with a constant flow of 500 μL/min. A linear gradient was employed starting with 5% B and reaching 50% B in 8 min and subsequently going to 99% B at 10 min.

### Identification of Novel Metagenomic Lasso Peptide BGCs

As a source for the detection of novel lasso peptide BGCs, the previously generated sequencing data of our metagenomic fosmid library, as well as the sequencing data of its corresponding HMW metagenomic DNA isolated from a soil sample collected in the Schönbuch forest (Germany), were used.^14^ The contigs of the metagenomic fosmid library were filtered first for sizes greater than 25 kb and, in a second step, for sizes greater than 5 kb to maximize the number of detectable BGCs. AntiSMASH^21^ version 5.0 was used to analyze contigs >25 kb, while antiSMASH version 7.0 was applied to those >5 kb. BGCs identified from contigs >5 kb were further examined for matches to similar known gene clusters within the MIBiG database^22^ using the KnownClusterBlast tool in antiSMASH. BGC 364 was identified from contigs >25 kb derived from direct metagenome sequencing. BGCs 468, 8976, and 9882 were selected from contigs >25 kb derived from the fosmid library sequencing. BGCs 38, 52, 54, and 89 were selected from contigs >5 kb derived from the same fosmid library data set.

### Prioritization and Annotation of BGCs for Heterologous Expression

To prioritize BGCs for expression, manual inspection was conducted to identify those with all of the biosynthetic genes in the same orientation. The minimal set of lasso peptide biosynthesis genes A, B, and C, as well as additional putative lasso peptide specific genes, were identified using the methodology described in our previous study.^14^ Briefly, all putative lasso peptide specific genes were identified using a combination of blastx^47^ and antiSMASH analysis, which included the comparison of the results to those of known lasso peptide BGCs. Lasso peptide A genes that were not detected by antiSMASH were identified by a manual inspection of the translated nucleotide sequence of candidate genes for the characteristics common to lasso peptide precursors.

### Refactoring and Cloning of Metagenomic Lasso Peptide BGCs

To isolate, refactor, and clone the lasso peptide BGCs, the lasso peptide specific genes, excluding those encoding isopeptidases, were amplified via PCR from the respective sources using manually designed primers with different types of overhangs (Supplemental Table S3). The overhangs served two purposes: (i) insertion of an *E. coli* optimized RBS (AGAGG­AGAAA­TTAACC)^30^ upstream of gene B (and C for BGC 89) for BGCs that lacked an apparent RBS upon manual inspection, and (ii) allowing for downstream assembly with the pET28a expression vector using an assembly technique based on Gibson Assembly.^48^

For the first group of BGCs (38, 52, 54, and 89), the minimal set of lasso peptide biosynthesis genes A, B, and C was amplified; in the case of BGC 38, the D gene and the gene encoding a tetratricopeptide repeat protein were also included. For the second group of BGCs (364, 468, 8976, and 9882), the minimal set of the lasso peptide biosynthesis genes A, B1, B2, and C as well as genes encoding phospho- or methyltransferases, where present, were amplified.

PCR amplifications were performed using a Q5 high-fidelity DNA polymerase kit (NEB). For each amplification, multiple 25 μL reactions were prepared. Fosmid DNA (150 ng per reaction) or metagenomic DNA (15 ng per reaction) served as templates. The reaction mixture (5 μL of 5 × Q5 reaction buffer, 5 μL of 5 × Q5 High GC Enhancer, 0.5 μL of 10 μM forward/reverse primer, 0.5 μL of 10 mM deoxynucleoside triphosphates (dNTPs), 3 μL of template DNA, 0.25 μL of Q5 high-fidelity DNA polymerase, and 10.25 μL of nuclease-free water) as well as the thermocycling conditions (98 °C for 30 s, 30 cycles of 98 °C for 10 s, annealing at 63 °C for 30 s, extension at 72 °C (20 s/kb), and a final extension at 72 °C for 2 min) followed the setup of our previous study.^14^ Five microliters of each reaction was used to assess amplification success on a 1% agarose gel. The remainder of each reaction was pooled, and amplicons were gel purified using the QIAquick Gel Extraction Kit (QIAGEN) following the manufacturer’s instructions.

To obtain the linearized expression vector ready for subsequent assembly with the amplified lasso peptide BGCs, the pET28a expression vector was digested with XbaI (Thermo Scientific) overnight and subsequently purified using a QIAquick PCR Purification Kit (QIAGEN). Purified lasso peptide gene amplicons with respective overhangs were assembled with the purified linearized pET28a vector using the NEBuilder HiFi DNA Assembly Master Mix (NEB), a cloning technique based on Gibson Assembly.^48^ The incubation time of the reaction was extended to 1 h at 50 °C. Five microliters of each assembly was used to transform 100 μL of *E. coli* DH10ß cells via electroporation (Bio-Rad MicroPulser Electroporator). Following recovery at 37 °C with shaking for 1 h, transformants were plated on LB agar containing 50 μg/mL of kanamycin (KAN) and incubated at 37 °C overnight. Five transformants per construct were selected for overnight culture in LB KAN and subjected to plasmid isolation via alkaline lysis. Isolated constructs were validated first by PCR and restriction digestion and then by sequencing using the whole plasmid sequencing service of Eurofins Genomics Europe. Obtained sequences were analyzed using Geneious (Version 9.1.8). All verified constructs were transferred to *E. coli* BL21 (DE3) via electroporation for expression studies.

### Culture Conditions and Extraction Procedures

For heterologous production, clones were cultivated in triplicates for each lasso peptide BGC. Culture conditions were selected using a combination of different procedures described in the literature^49,50^ and were as follows. Precultures (5 mL of LB KAN) were inoculated with each of the *E. coli* BL21 (DE3) clones carrying the respective lasso peptide BGC construct, as well as a clone carrying the empty expression vector (negative control), and were grown at 37 °C with shaking overnight (ON). These cultures were used to inoculate 100 mL of M9 minimal medium^49^ supplemented with 50 mg/L of each proteinogenic amino acid and KAN to an initial OD_600_ of 0.01. Cultures were grown at 37 °C with shaking to an OD_600_ of ∼0.45 and then shifted to 20 °C. After 40 min, IPTG was added to a final concentration of 1 mM to induce expression. Following 3 days of incubation, cells and supernatants were harvested and extracted separately. Extraction of cell pellets and culture media as well as subsequent analysis of extracts were performed as described in detail in our previous study.^14^

### Analysis of Environmental Distribution and Phylogeny of BGC 89

To investigate the environmental distribution of BGC 89, the corresponding contig was analyzed using the BGC Atlas web platform.^35^ The taxonomy of all eight BGCs was investigated by comparing them to entries in the Genome Taxonomy Database (GTDB, v220)^36^ using mmseqs (v 16.747c6)^51^ with the easy-taxonomy command and default parameters. The relationship of BGC 89 to other BGCs in the NCBI database was assessed using the cblaster web platform.^41^ Results were visualized using the clinker web platform.^42^ For this, contigs corresponding to the top five cblaster hits, along with the contig corresponding to the closest match with the GTDB database, were downloaded, and the lasso peptide BGCs were extracted using Geneious software. These BGCs, along with BGC 89, were then subjected to comparative analysis using a clinker. To compare the amino acid sequence of metanodin to that of the lasso peptide predicted to be produced by the second closest matching BGC, the corresponding contig was analyzed with antiSMASH to identify the precursor peptide, which was previously not annotated.

## Supplementary Material



## Data Availability

antiSMASH results are available for download from 10.5281/zenodo.16679566. The soil metagenome sequencing data is stored in the NCBI Sequence Read Archive (SRA) and is accessible under BioProject identifier PRJNA717813 (Runs: SRR14085589, SRR14085590, SRR14085591). The corresponding assembly is stored in the European Nucleotide Archive (ENA) under the accession ERZ28533175. The fosmid library sequencing data is accessible under BioProject identifier PRJNA799808 (NCBI SRA). The corresponding assembly is stored in the ENA under the accession ERZ28533575. All further relevant data generated or analyzed during this study are included in this published article and its Supporting Information.

## Notes

Total ion chromatograms of pellet extracts from *E. coli* pET28a and *E. coli* pET28a_BGC89 (Figure S1); Fragmentation of the metanodin macrolactam ring yielding nine distinct linearized peptides (Figure S2); Blastx results, gene sizes, and antiSMASH annotations of metagenomic lasso peptide BGCs (Supplemental Tables S1 and S2); Primers with overhangs for amplification, refactoring, and cloning of metagenomic lasso peptide BGCs (Supplemental Table S3) (PDF)
