# Supplementary material for: Discovery and Heterologous Expression of the Soil Metagenome-Derived Lasso Peptide Metanodin with an Unprecedented Ring Structure
Source: J Nat Prod. 2025 Oct 27;88(11):2625–34. doi: 10.1021/acs.jnatprod.5c00970 (PMC12670498; doi:10.1021/acs.jnatprod.5c00970)
Supplement: Supplementary file 1 [file np5c00970_si_001.pdf]

Supporting Information

Discovery and Heterologous Expression of the Soil Metagenome-Derived Lasso Peptide Metanodin with an Unprecedented Ring Structure

Timo Negri [a, b, §], Giovanni Andrea Vitale [c, d, §], Martina Adamek [a, b], Caner Bağcı [a, b, e], Julian D. Hegemann [f, g], Daniel Petras [c, h, i], Chambers C. Hughes [b, d, i,*], and Nadine Ziemert [a, b, e,*]

[a] Translational Genome Mining for Natural Products, Interfaculty Institute of Microbiology and Infection Medicine (IMIT), University of Tübingen, Auf der Morgenstelle 24, 72076 Tübingen, Germany

[b] German Centre for Infection Research (DZIF), Partner Site Tübingen, 72076 Tübingen, Germany

[c] Functional Metabolomics Laboratory, Interfaculty Institute of Microbiology and Infection Medicine (IMIT), University of Tübingen, Auf der Morgenstelle 24, 72076 Tübingen, Germany

[d] Department of Microbial Bioactive Compounds, Interfaculty Institute of Microbiology and Infection Medicine (IMIT), University of Tübingen, Auf der Morgenstelle 28, 72076 Tübingen, Germany

[e] Institute for Bioinformatics and Medical Informatics (IBMI), University of Tübingen, Sand 14, 72076 Tübingen, Germany

[f] Institute of Pharmaceutical Biology, Technische Universität Braunschweig, 38106 Braunschweig, Germany

[g] Center of Pharmaceutical Engineering (PVZ), Technische Universität Braunschweig, 38106 Braunschweig, Germany

[h] Department of Biochemistry, University of California Riverside, CA 92507, USA

[i] Cluster of Excellence EXC 2124: Controlling Microbes to Fight Infection, University of Tübingen, 72076 Tübingen, Germany

[*] E-Mail: chambers.hughes@uni-tuebingen.de

[*] E-Mail: nadine.ziemert@uni-tuebingen.de

[§] T.N. and G.A.V. contributed equally to this work

Table of contents

Figure S1. Total ion chromatograms of pellet extracts from *E. coli* P3

Figure S2. Mass spectrometry and HCD analyses of metanodin P4

Table S1. Identification of putative lasso peptide genes of Group I BGCs P5

Table S2. Identification of putative lasso peptide genes of Group II BGCs P7

Table S3. Primers with overhangs for amplification, refactoring and cloning of metagenomic lasso peptide BGCs P10

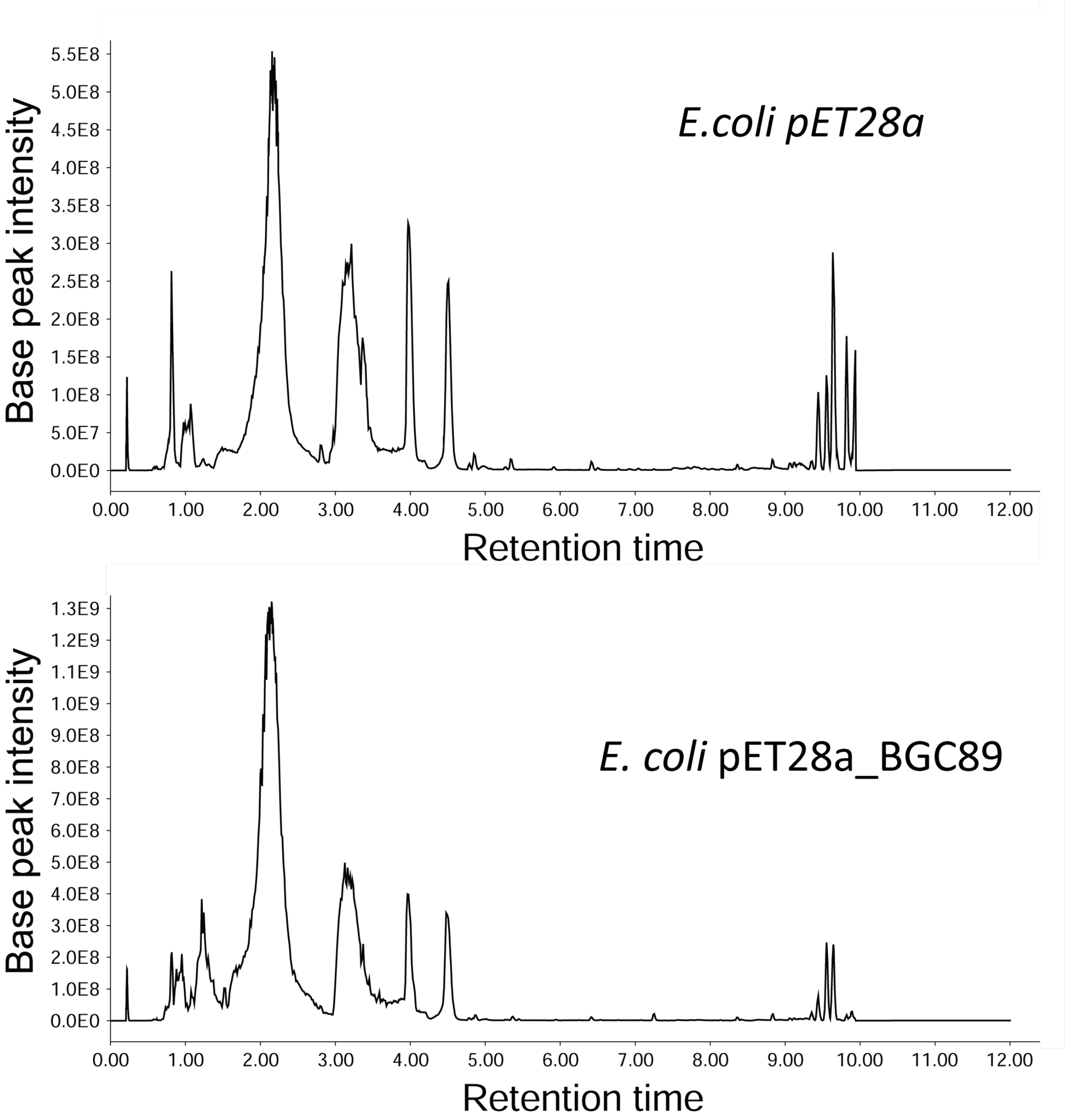

Figure S1. Total ion chromatograms of pellet extracts from *E. coli* pET28a and *E. coli* pET28a_BGC89, shown in the upper and lower panels respectively.

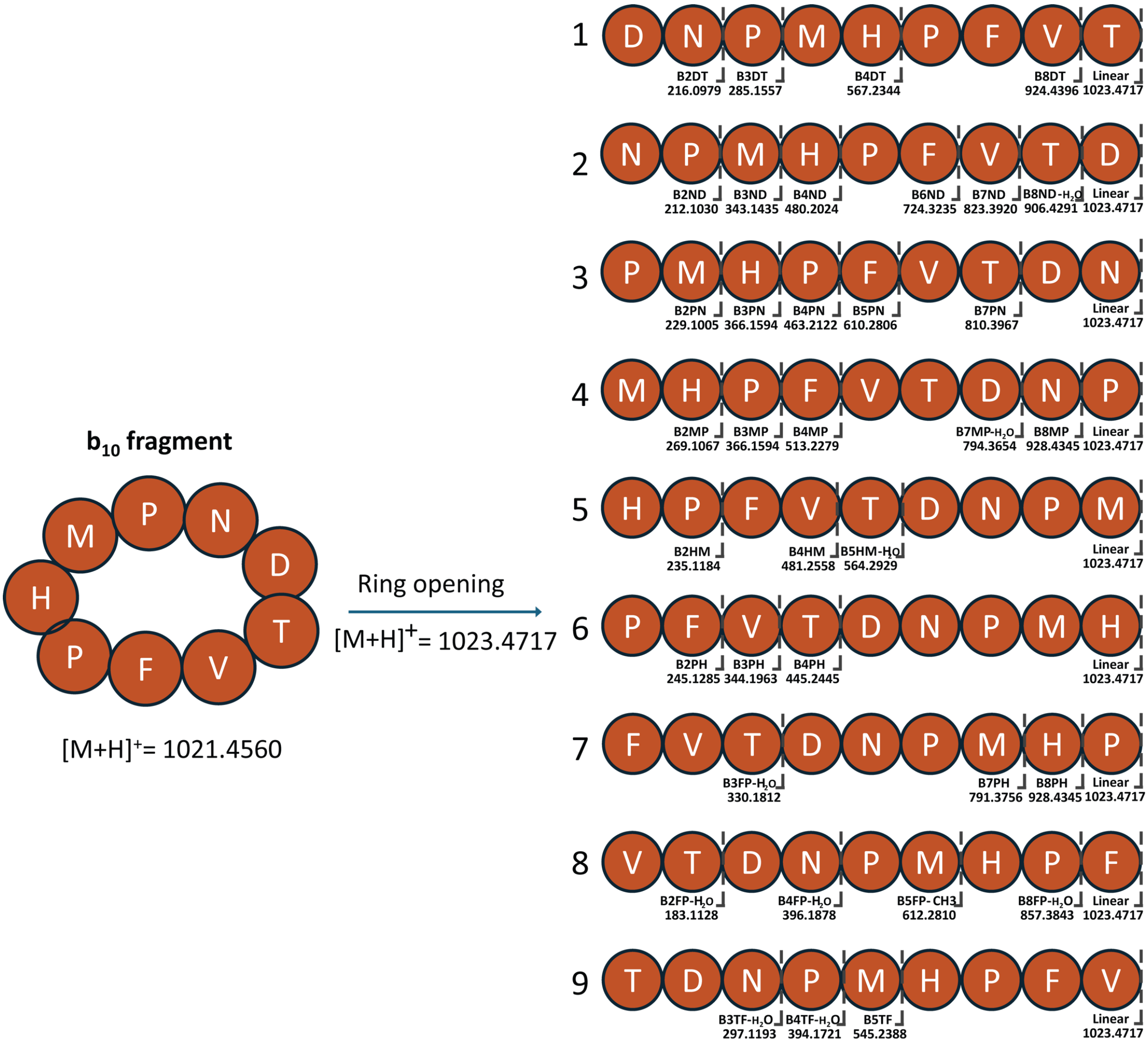

Figure S2. Mass spectrometry and HCD analyses confirm the macrolactam ring sequence of metanodin. Fragmentation of the metanodin macrolactam ring (initiated at b_{10}) yields nine distinct linearized peptides (1–9) and a total of 35 diagnostic b-ions.

Table S1. Identification of putative lasso peptide genes of BGCs 38, 52, 54 and 89 using Blastx results, gene sizes and antiSMASH annotations.

**Metagenomic lasso peptide BGC 38**						
**Putative lasso peptide gene**	**Blastx result***	**Query Cover**	**E Value**	**Percent Identity**	**Gene size (bp)**	**antiSMASH annotation**
D	ABC transporter six-transmembrane domain-containing protein [*Blastocatellia bacterium*]	90%	6,00E-85	48.45%	972	-
A1	No significant similarity found				198	predicted lassopeptide
B	lasso peptide biosynthesis B2 protein [*Armatimonadota bacterium*]	91%	1,00E-53	42.68%	786	PF13471
C	asparagine synthase-related protein [*Hydrococcus rivularis*] (2nd Blast result)	95%	3,00E-150	41.36%	1920	Asn_synthase
ctg38_17	tetratricopeptide repeat protein [*Gemmataceae bacterium*]	66%	4,00E-10	48.60%	486	-
A2	caspase family protein [*Gemmataceae bacterium*]	97%	1,00E-10	48.21%	174	-
**Metagenomic lasso peptide BGC 52**						
**Putative lasso peptide gene**	**Blastx result**	**Query Cover**	**E Value**	**Percent Identity**	**Gene size (bp)**	**antiSMASH annotation**
A	No significant similarity found				93	predicted lassopeptide
B	lasso peptide biosynthesis B2 protein [*Steroidobacteraceae bacterium*]	92%	1,00E-57	48.26%	735	PF13471
C	asparagine synthase-related protein [*Steroidobacteraceae bacterium*]	98%	0.0	54.38%	1887	Asn_synthase
Isopeptidase	prolyl oligopeptidase family serine peptidase [*Steroidobacteraceae bacterium*]	99%	0.0	54.17%	2100	Peptidase_S9

Metagenomic lasso peptide BGC 54						
**Putative lasso peptide gene**	**Blastx result**	**Query Cover**	**E Value**	**Percent Identity**	**Gene size (bp)**	**antiSMASH annotation**
A1	MAG: hypothetical protein DI624_00395 [*Brevundimonas sp.*]	98%	9,00E-21	93.18%	135	predicted lassopeptide
A2	hypothetical protein [*Brevundimonas sp.*]	98%	2,00E-20	100.00%	129	-
B	lasso peptide biosynthesis B2 protein [*Brevundimonas sp.*]	94%	1,00E-101	99.54%	687	PF13471
C	asparagine synthase-related protein [*Brevundimonas sp.*] (2nd Blast result)	100%	0.0	92.66%	1719	Asn_synthase
Isopeptidase	prolyl oligopeptidase family serine peptidase [*Alphaproteobacteria bacterium*] (3rd Blast result)	94%	0.0	50.52%	2448	Peptidase_S9

Metagenomic lasso peptide BGC 89						
**Putative lasso peptide gene**	**Blastx result**	**Query Cover**	**E Value**	**Percent Identity**	**Gene size (bp)**	**antiSMASH annotation**
A	No significant similarity found				99	-
B	lasso peptide biosynthesis B2 protein [*Steroidobacteraceae bacterium*]	80%	5,00E-52	41.83%	939	PF13471
C	asparagine synthase C-terminal domain-containing protein [*Steroidobacteraceae bacterium*]	99%	0.0	46.28%	1857	Asn_synthase
Isopeptidase	prolyl oligopeptidase family serine peptidase [*Rhizomicrobium sp.*]	95%	0.0	49.62%	2466	Peptidase_S9

* Blastx results include Query Cover, E Value and Percent Identity

Table S2. Identification of putative lasso peptide genes of BGCs 364, 468, 8976 and 9882 using Blastx results, gene sizes and antiSMASH annotations.

**Metagenomic lasso peptide BGC 364**						
**Putative lasso peptide gene**	**Blastx result***	**Query Cover**	**E Value**	**Percent Identity**	**Gene size (bp)**	**antiSMASH annotation**
B1	PqqD family protein [*Terriglobales bacterium*]	99%	2,00E -53	90.11%	276	PF05402
Phosphotransferase	phosphotransferase [*Terriglobales bacterium*]	100%	0.0	92.21%	1389	-
Isopeptidase	prolyl oligopeptidase family serine peptidase [*Terriglobales bacterium*]	100%	0.0	86.88%	2061	Peptidase_S9
B2	lasso peptide biosynthesis B2 protein [*Candidatus Acidiferrum sp.*]	99%	2,00E -76	87.10%	375	PF13471
C	asparagine synthase-related protein [*Candidatus Acidiferrum sp.*]	96%	0.0	71.98%	1923	Asn_synthase
A	No significant similarity found				150	-
A	hypothetical protein [*Candidatus Acidiferrum sp.*]	98%	0.001	48.98%	150	-
**Metagenomic lasso peptide BGC 468**						
**Putative lasso peptide gene**	**Blastx result**	**Query Cover**	**E Value**	**Percent Identity**	**Gene size (bp)**	**antiSMASH annotation**
D	ABC transporter ATP-binding protein [*Candidatus Acidiferrales bacterium*]	98%	0.0	70.93%	1740	ABC transporter related protein
B1	PqqD family protein [*Verrucomicrobiia bacterium*]	99%	3,00E -56	97.80%	276	PF05402
Phosphotransferase	phosphotransferase [*Verrucomicrobiia bacterium*]	100%	0.0	98.29%	1404	-
Isopeptidase	prolyl oligopeptidase family serine peptidase [*Verrucomicrobiia bacterium*]	100%	0.0	99.56%	2037	Peptidase_S9
B2	lasso peptide biosynthesis B2 protein [*Verrucomicrobiia bacterium*]	99%	4,00E -85	98.39%	375	PF13471

C	asparagine synthase-related protein [*Candidatus Acidiferrales bacterium*]	100%	0.0	85.76%	1902	Asn_synthase
A	hypothetical protein [*Terriglobales bacterium*]	98%	2,00E -05	55.32%	144	-
**Metagenomic lasso peptide BGC 8976**						
**Putative lasso peptide gene**	**Blastx result**	**Query Cover**	**E Value**	**Percent Identity**	**Gene size (bp)**	**antiSMASH annotation**
B1	PqqD family peptide modification chaperone [*Vicinamibacterales bacterium*]	80%	7,00E -31	67.95%	291	PF05402
B2	lasso peptide biosynthesis B2 protein [*Candidatus Kapaibacterium sp.*]	94%	4,00E -28	52.24%	429	lassopeptide: PF13471
M	FkbM family methyltransferase [*Acidobacteriota bacterium*] (5th Blast result)	96%	7,00E -53	35.06%	966	-
C	lasso peptide isopeptide bond-forming cyclase [*Terriglobia bacterium*]	100%	0.0	56.07%	1827	Asn_synthase
A	No significant similarity found				159	-
Phosphotransferase	serine/threonine protein kinase [*Terriglobia bacterium*]	98%	7,00E -95	51.10%	957	-
**Metagenomic lasso peptide BGC 9882**						
**Putative lasso peptide gene**	**Blastx result**	**Query Cover**	**E Value**	**Percent Identity**	**Gene size (bp)**	**antiSMASH annotation**
B1	PqqD family protein [*Candidatus Acidiferrales bacterium*]	99%	8,00E -55	93.41%	276	PF05402
Phosphotransferase	phosphotransferase [*Candidatus Acidiferrales bacterium*]	99%	0.0	84.75%	1392	-
Isopeptidase	prolyl oligopeptidase family serine peptidase [*Candidatus Acidiferrales bacterium*]	100%	0.0	89.68%	2037	Peptidase_S9
B2	lasso peptide biosynthesis B2 protein [*Candidatus Acidiferrales bacterium*]	94%	5,00E -82	95.16%	396	PF13471

C	asparagine synthase-related protein [*Candidatus Acidiferrales bacterium*]	99%	0.0	65.79%	1938	Asn_synthase
A	hypothetical protein [*Candidatus Acidiferrum sp.*]	98%	3,00E -06	54.17%	147	-

* Blastx results include Query Cover, E Value and Percent Identity

Table S3. Primers with overhangs for amplification, refactoring and cloning of metagenomic lasso peptide BGCs. Overhang sequences for assembly of the fragments in red. Ribosome binding site sequences in bold.

**Primer**	**Sequence**	**Targeted BGC**
Cl38_OV_fw	GAGCGGATAACAATTCCCCTCAATACTCATCGCAAGCGGC	38
Cl38_OV_rv	AAAGTTAAACAAAATTATTTGGCCAGGATGAGTTGCTTCT	
Cl52_OV_fw	GAGCGGATAACAATTCCCCTCCGGTTTGAACGTTGTGTCC	52
Cl52_A_RBS_OV_rv	**GGTTAATTTCTCCTCT**TTAGACGTGGAATTGGTGAG	
Cl52_OV_B_fw	CTAA**AGAGGAGAAATTAACC**ATGCCGGAAGCAAAGTATTT	
Cl52_OV_rv	AAAGTTAAACAAAATTATTTGGAACAAGCTTCGCGAGAT	
Cl54_OV_fw	GAGCGGATAACAATTCCCCTAAATACACTTCCGAGGGCGC	54
Cl54_A_RBS_OV_rv	**GGTTAATTTCTCCTCT**TCAGCGCGCCGCGGACGGCT	
Cl54_OV_B_fw	CTGA**AGAGGAGAAATTAACC**TTGCTGGTTCCTGAGCCTCT	
Cl54_OV_rv	AAAGTTAAACAAAATTATTTACGCTACATCGACGCTAGTC	
Cl89_OV_fw	GAGCGGATAACAATTCCCCTGACCGTAGTCACGTCGATCG	89
Cl89_A_RBS_OV_rv	**GGTTAATTTCTCCTCT**TTAAATATTGAGGCCATGGT	
Cl89_OV_B_fw	TTAA**AGAGGAGAAATTAACC**GTGTGCGGCACAGGCCGCACACCTCCTCCAAGCTCG	
Cl89_OV_B_RBS__rv	**GGTTAATTTCTCCTCT**CTAAACGCAAAGAAGCGGAT	
Cl89_RBS_OV_C_fw	TTAG**AGAGGAGAAATTAACC**ATGTATCGCTACGTCGTACT	
Cl89_OV_rv	AAAGTTAAACAAAATTATTTTCGAGGTGAAGTGACCATCG	
Cl364_OV_RBS_B1_fw	GAGCGGATAACAATTCCCCT**AGAGGAGAAATTAACC**ATGTTCCGAATATCCGACAC	364
Cl364_Phos_rv	TCAGTTCAGGCACCGTTCGC	
Cl364_OV_B2_fw	GCGAACGGTGCCTGAACTGAATCGGAATACAAAGGAGAGC	
Cl364_Prc_OV_rv	AAAGTTAAACAAAATTATTTCGATTTCCGCCTGCTGATTG	
Cl468_OV_RBS_B1_fw	GAGCGGATAACAATTCCCCT**AGAGGAGAAATTAACC**ATGTTCACACTAGCCGACAG	468
Cl468_Phos_rv	TCAGCGCAGGCATGGTTCGC	

Cl468_OV_B2_fw	GCGAACCATGCCTGCGCTGAAGGAGGCTGGTGATGCGGTT	
Cl468_OV_Prc_rv	AAAGTTAAACAAAATTATTTCGGTTGAATCTTGCGTCTTT	
Cl8976_OV_RBS_B1_fw	GAGCGGATAACAATTCCCCT**AGAGGAGAAATTAACC**ATGACGAACCTGGTGGTCTC	8976
Cl8976_OV_ProtKin_rv	AAAGTTAAACAAAATTATTTGCCTGGTCGTGATTCTCCAT	
Cl9882_OV_RBS_B1_fw	GAGCGGATAACAATTCCCCT**AGAGGAGAAATTAACC**ATGTTCCGAGTCTCCGACAC	9882
Cl9882_Phos_rv	TCAGTCGAGGCACAGTTCGC	
Cl9882_OV_B2_fw	GCGAACTGTGCCTCGACTGAGAGGAGGGAGTTCAGCCATG	
Cl9882_OV_Prc_rv	AAAGTTAAACAAAATTATTTACAAAATGCTGTCCCTCTTG	
